# Maintenance of Physical Activity and Exercise Capacity After Rehabilitation in Coronary Heart Disease: A Randomized Controlled Trial

**DOI:** 10.5539/gjhs.v6n6p198

**Published:** 2014-07-29

**Authors:** Hassan Okati Aliabad, Mohammadreza Vafaeinasab, Mohammad Ali Morowatisharifabad, Seyed Alireza Afshani, Mahdieh Ghanbari Firoozabadi, Seyed Khalil Forouzannia

**Affiliations:** 1 Department of Health Education and Promotion, Shahid Sadoughi University of Medical Sciences, Yazd, Iran; 2 Cardiovascular Research Center, Shahid Sadoughi University of Medical Sciences, Yazd, Iran; 3 Faculty of Social Sciences, Yazd University, Yazd, Iran; 4 Tehran Heart Center, Tehran University of Medical Sciences, Tehran, Iran

**Keywords:** physical activity, exercise capacity, coronary heart disease, health action process approach, family support

## Abstract

**Background and Objectives:**

Physical activity is one of the core components in cardiac rehabilitation and secondary prevention programs. This study investigated the effect of an intervention based on the health action process approach (HAPA) together with family support in the maintenance of physical activity and exercise capacity in coronary heart disease after discharge from rehabilitation.

**Method and Materials:**

In this randomized controlled trial, 96 patients with coronary heart disease were randomly assigned to control and intervention groups at the end of a rehabilitation program at Afshar Hospital, Yazd, Iran. HAPA Constructs and family support using a self-reported questionnaire and maximal oxygen uptake through a treadmill exercise test were measured prior to and 4 months after the intervention.

**Results:**

HAPA-based intervention together with family support increased scores of HAPA constructs and family support in the intervention group compared with the control group. The results showed that physical activity and exercise capacity in the intervention group was significantly higher than the control group after the intervention.

**Conclusion:**

HAPA-based intervention together with family support can be a useful tool for maintenance of physical activity and exercise capacity in coronary heart disease.

## 1. Introduction

**Figure 1:**
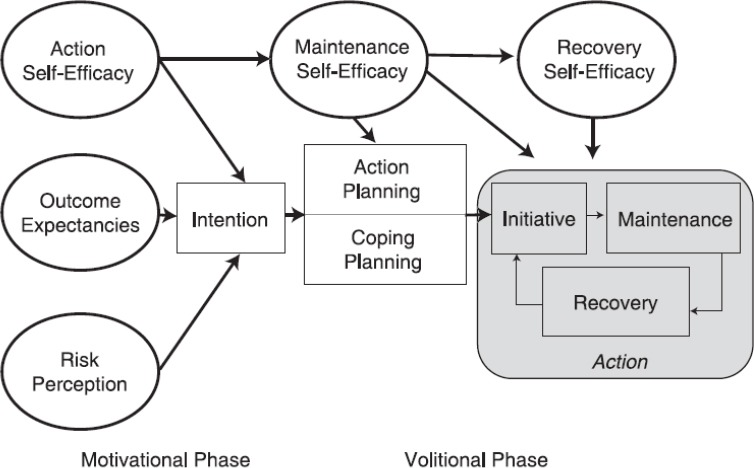
The diagram of the Health Action Process Approach

Cardiovascular diseases (CVDs) are the major cause of mortality all over the world including Iran. Worldwide, around 30% of all mortalities are due to CVDs, among which approximately 42% are due to coronary heart disease (CHD) (WHO, 2013; Naghavi, 2005). Patients suffering from CHD should be regularly referred to cardiac rehabilitation and should be put under exercise programs after serious cardiac problems, and they should be encouraged to participate in such programs (Lavie & Milani, 2011). As the American Heart Association reports, exercise is one of the core components of cardiac rehabilitation and secondary prevention programs (Balady et al., 2007). Regular exercise, physical activity, and maintenance a high level of Cardio respiratory fitness are considered necessary elements in CVD prevention and treatment and play an important role in reducing the risk of suffering from CHD in primary and secondary prevention (Archer & Blair, 2011; Lavie, Thomas, Squires, Allison, & Milani, 2009). All over the World, a lack of physical activity causes 6% of the disease load of CHD (Lee et al., 2012). After a cardiac rehabilitation program, sedentary lifestyle has a negative impact on the major risk factors (Freyssin, Blanc, Verkindt, Maunier, & Prieur, 2011). Exercise capacity is the strongest predictor of mortality compared with the other risk factors (Myers et al., 2002). Exercise maintenance is one of the factors which improve the quality of life and physical activity level (Izawa et al., 2004). Research shows that regular exercise maintenance after rehabilitation typically decreases with the passage of time. While 83% of participants started an exercise during one month after rehabilitation, after one month, one third stopped exercising and less than 37% followed the exercise program for one year (Dolansky, Stepanczuk, Charvat, & Moore, 2010; Moore, Ruland, Pashkow, & Blackburn, 1998). Interventions including patient education are effective in increasing physical activity (Conn, Hafdahl, Brown, & Brown, 2008). Interventions which use behavioral change methods to increase physical activity during and after rehabilitation, and for the patients without rehabilitation, show that such interventions increase physical activity (Ferrier, Blanchard, Vallis, & Giacomantonio, 2011). Also interventions using combined methods after cardiac rehabilitation, have led to better results (Bock et al., 1997). Findings show that the strongest variables related to maintenance of physical activity are beliefs relevant to abilities, motivations, and goals. However, other variables such as post-intentional constructs or self-regulation processes are also effective in the maintenance of physical activity (Amireault, Godin, & Vézina-Im, 2012). HAPA is a health behavior change model, which distinguishes between pre-intentional motivational processes leading to behavioral intention and post-intentional volitional processes leading to actual health behavior. In the motivational phase risk perception, positive outcome expectancies and perceived self-efficiency cause intention formation. When the intention is formed, to translate intention to behavior in the Volitional Phase, post-intentional factors such as planning and self-efficiency cause action initiation and maintenance (Schwarzer, 2008). The diagram of the Health Action Process Approach is illustrated in Figure 1.

If planning is integrated into the rehabilitation programs, it leads to sustainable recovery (Reuter, Ziegelmann, Lippke, & Schwarzer, 2009). Thus, it must be recommended to individuals who intend to change their behavior to have realistic feasible plans to achieve success (Scholz, Sniehotta, Schüz, & Oeberst, 2007). A combination of action planning and coping planning has more effects on health behavior and physical activity change. Volitional interventions should include both coping and action planning (Araújo-Soares, McIntyre, & Sniehotta, 2009; Wiedemann, Lippke, Reuter, Ziegelmann, & Schwarzer, 2011). In those with a higher intention to do exercise, planning mediates behavior intention relation. Thus, interventions in exercise behavior promotion must take both intention and planning into account (Conner, Sandberg, & Norman, 2010; Wiedemann, Schüz, Sniehotta, Scholz, & Schwarzer, 2009). Coping planning plays an important role in long-term exercise maintenance (Ziegelmann, Lippke, & Schwarzer, 2006). Various kinds of self-efficiency are effective during and after a cardiac rehabilitation (Rodgers, Murray, Selzler, & Norman, 2013). Maintenance self-efficiency is important for those who are in maintenance phase and do exercise according to the recommended level. And recovery self-efficiency is useful for those who are subject to lapse risk and restart their physical activity after a failure (Luszczynska & Sutton, 2006; Scholz, Sniehotta, & Schwarzer, 2005). In the treatment of chronic diseases, those interventions in which family members participate, positively impact on both patient and family members (Hartmann, Bazner, Wild, Eisler, & Herzog, 2010). Individuals’ physical activity planning is often affected by those in a social setting who support these behaviors (Molloy, Dixon, Hamer, & Sniehotta, 2010). Also, social psychological factors affect exercise tolerance in cardiac patients (Fraser & Rodgers, 2010). Thus, the interventions are necessary to increase understanding positive social resources (Barth, Schneider, & von Kanel, 2010). Findings show that different perceptions of social support are related to differences self-efficiency and quality of life among maintainers of cardiac rehabilitation exercise and social support affects physical activity like direct prerequisite of self-efficiency and self-regulation (Anderson, Wojcik, Winett, & Williams, 2006; Woodgate, Brawley, & Shields, 2007). According to the above mentioned issues and shortage of studies done regarding post-rehabilitation and lack of studies based on HAPA in Iran, the present study investigates whether HAPA-based intervention together with family support increases the scores of HAPA constructs and family support in intervention group compared with the control group. Also, this study investigates whether HAPA-based intervention together with family support is effective compared with Afshar Hospital, Yazd, Iran, ordinary training in maintenance of physical activity and exercise capacity in CHD after discharge from the rehabilitation.

## 2. Method

**Figure 2:**
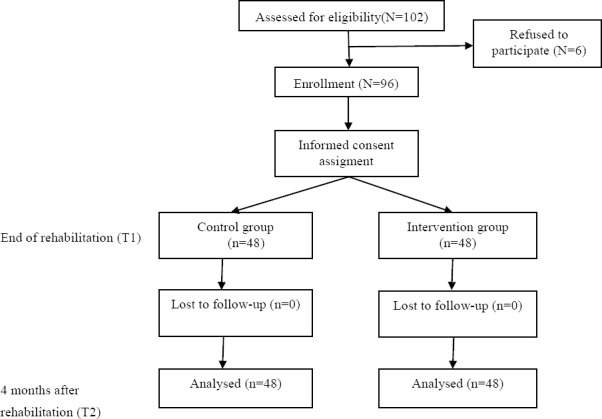
Flow diagram of participants.

In this randomized controlled trial (RCT), 96 CHD who completed rehabilitation in Afshar hospital, Yazd, Iran, participated. The Ethical committee of Shahid Sadoughi university of medical sciences validated the study method and protocols. Inclusion criteria included having CHD, reading and writing literacy, completing a rehabilitation program in Afshar hospital, Yazd, Iran, and presenting the Informed consent form to participate in the project. Exclusion criteria included illiteracy and serious psychological problems. Using obtained data from a pilot study (power=80%, P-value=. 05), patients were selected at the end of their rehabilitation program and randomly assigned to control and intervention groups according to a random number list obtained from Generate Random Numbers software 1.0. The participant flow throughout the study is illustrated in Figure 2.

Patients were not informed about their assignment to the control or the intervention groups. They first filled the informed consent form and a questionnaire in which HAPA social cognitive factors, including risk perception, outcome expectancies, task self-efficiency, behavioral intention, action planning, coping planning, recovery self-efficiency, family support and past physical activity (before chronic heart problem) were examined. Demographic information was recorded from the participants’ profiles. In addition, at the end of rehabilitation, exercise test was run on both the control and intervention groups and the amount of Maximal oxygen uptake showing their exercise capacity was examined. The intervention group received HAPA-based training, a booklet designed based on HAPA, and the control group received the Afshar hospital, Yazd, Iran, ordinary training and their booklet. Training was done in three sessions in the form of individual discussions with each participant by a booklet designed based on HAPA. In the first session, recognized effective motivational phase factors of the model, including risk perception, outcome expectancies, task self-efficiency, and intention were discussed. In the booklet, information about the relationship between physical activity and health in cardiac patients and benefits of physical exercise for them, also persuasive messages and reminders successful experiences of the participants during rehabilitation to increase their self-efficiency, was given to the participants. They were encouraged to decide on continuing exercise after rehabilitation. In addition, they were asked to question their potential questions for the ambiguity removal. In the second session, volitional phase factors were discussed. The participants were asked if they have any plan to continue their physical activity after rehabilitation, if yes, they were asked to write about their action plan, including when, where, how and how often they do physical activity and with whom, in one of their booklet pages, which was designed in the form of a table. They were also asked to write, whether they have any plan overcome potential barriers while doing exercise after rehabilitation. If yes, they were to write the potential barriers and the solutions in another page, again in the form of a table. These issues were discussed with them and the importance of planning to achieve the goal was recollected to them. On another page of the booklet, the potential barriers obtained by a pilot study and the solutions were provided to them in the form of a table. To increase maintenance self-efficiency, the patients were encouraged to keep with their physical activity program, write specific and realistic goals and not to be disappointed with the problems in their program, and they were asked to write about their feeling after physical activity so that they would gain the required self-efficiency. To increase their recovery self-efficiency, they were encouraged not to be anxious about the missed days if for one reason or another, they could not continue their exercise for a few days, and not to lose their motivation and return to their program. To involve families and increase social support, the patient’s spouse or the most significant person in their life was invited to participate in the last training session and required strategies to increase social support were discussed with them. These strategies included sharing the participants’ exercise goals; encouraging and supporting them, accompanying them while doing physical activity and doing some affairs delegated to the participants. In addition, the participants were asked to involve their family members in their physical activity program. At the end, an educational booklet designed based on HAPA was given to them. The control group received the Afshar Hospital, Yazd, Iran, ordinary training, and their booklet in which information about factors disposing CHD and benefits of physical activity and various kinds of activity was given. The type of exercise recommended to participants in the control group and intervention group was based on their exercise test before discharge from the rehabilitation that was prescribed to them by a rehabilitation specialist and included aerobic, resistance, flexibility and balance exercises. 4 months after rehabilitation, the participants were invited again to the hospital and both groups filled a questionnaire in which HAPA social cognitive factors, family support and past four-month physical activity were examined. In addition, exercise test was run on both groups and their amount of maximal oxygen uptake was compared with each other. The patients’ demographic information including age, sex, employment status, education level and disease diagnosis were recorded from their profile.

### 2.1 Measuring

Data collection tools used in this study were questionnaire and treadmill exercise test both run in the form of pretest and posttest. In the questionnaire, HAPA social cognitive factors, including risk perception, outcome expectancies, task self-efficiency, behavioral intention, action planning, coping planning, recovery self-efficiency, and family support were assessed. All items had a 4-point Likert scale response range from 1 (not at all true) to 4 (exactly true). Validity and reliability of the questionnaires were measured through a pilot study. Internal consistency of the questionnaires was evaluated using Cronbach’s alpha.

#### 2.1.1 Risk Perception

Risk perception was measured by 5 items assessing vulnerability from a sedentary lifestyle with the stem if I have a sedentary lifestyle… followed by 5 statements that indicate future problems, such as, cardiovascular problems would suffer again. Summing all items up, risk perception total score was calculated from 5 to 20 (Cronbach’s alpha =. 78).

#### 2.1.2 Outcome Expectancies

Outcome expectancies were measured by10 items, 6 of which assessed positive outcome expectancies of exercise (pros) and 4 of which, negative outcome expectancies (cons) with the stem If I want to exercise regularly… followed by items of pros and cons. Pros were measured by 6 items such as, then I would be less vulnerable to disease. Cons were measured by 4 items such as, then I do not have enough time for other tasks. Negative outcome expectancies were recorded again and summing pros and cons items up, outcome expectancies total score was calculated from 10 to 40 (Cronbach’s alpha =. 77).

#### 2.1.3 Task Self-Efficiency

Task self-efficiency was measured by 3 items using the same techniques as Scholz et al., (2005), for example, ‘‘I am confident that I can adjust my life to a physically active lifestyle.’’ Task Self-efficiency total score from 3 to 12 was calculated summing item scores up (Cronbach’s alpha =. 72).

#### 2.1.4 Maintenance Self-Efficiency

Maintenance self-efficiency was measured by 4 items using the same techniques as Luszczynska and Sutton, (2006), for example, “I am confident that I am able to do rehabilitation exercises regularly even if I do not see any positive effects of the exercises.” Maintenance self-efficiency total score from 4 to 16, was calculated summing item scores up (Cronbach’s alpha =. 77).

#### 2.1.5 Recovery Self-Efficiency

Recovery self-efficiency was measured by 4 items using the same techniques as Luszczynska and Sutton, (2006), for example, “I am confident that I am able to resume the regular performance of exercises (after giving them up) even if I feel weak after illness period.” Recovery self-efficiency total score from 4 to 16, was calculated summing item scores up (Cronbach’s alpha =. 78).

#### 2.1.6 Behavioral Intention

Behavioral intention was measured by 4 items using the same techniques as Sniehotta, Schwarzer, Scholz, and Schüz, (2005), for example, “I intend to adhere to the exercise regime prescribed to me during the rehabilitation.” Intention total score from 4 to 16, was calculated summing item scores up (Cronbach’s alpha =. 70).

#### 2.1.7 Action Planning

Action planning was measured by 5 items addressing when, where, how, how often, and with whom to exercise using the same techniques as Lippke, Fleig, Pomp, and Schwarzer, (2010), for example, “For the month after the rehabilitation, I have already planned on which days of the week I will be physically active.” Action planning total score from 5 to 20, was calculated summing item scores up (Cronbach’s alpha =. 90).

#### 2.1.8 Coping Planning

Coping planning was measured by 5 items using the same techniques as Sniehotta, et al. (2005), for example, “I have made a detailed plan regarding what to do if something interferes with my plans.” Coping planning total score from 5 to 20, was calculated summing item scores up (Cronbach’s alpha =. 88).

#### 2.1.9 Family Support

Family support was measured by 5 items using the same techniques as Schwarzer, Lippke, and Luszczynska (2011), for example, “My family have encouraged me to perform my planned activities.” Family support total score from 5 to 25, was calculated summing item scores up (Cronbach’s alpha =. 88).

#### 2.1.10 Physical Activity

Physical activity was measured based on a modified version of the Godin Leisure-Time Exercise Questionnaire (Godin & Shephard, 1985). The participants were asked to say that whether in the last month before cardiac problem in pre-test and in the last month in post-test, how often a week and in what duration, they had strenuous (in which their heart rate has increased and they have perspired), moderate (in which they have not got tired or have perspired a bit) or mild exercise (in which they have not had much effort and have not perspired). Then, answers in each of three categories of exercise converted into metabolic equivalent (MET) minutes by multiplying the weekly minutes of mild exercise by 2.5 METs, moderate exercise by the 4.0 METs, and strenuous exercise by 7.5 METs. The physical exercise total score was calculated summing the scores of all three exercise categories up (Cronbach’s alpha =. 79). This questionnaire has been used in previous studies (Fleig, Lippke, Pomp, & Schwarzer, 2011; Lippke, Fleig, Pomp, & Schwarzer, 2010; Plotnikoff et al., 2007).

#### 2.1.11 Maximal Oxygen Uptake

Maximal oxygen uptake was measured based on the Bruce protocol (Bruce, 1971) using treadmill exercise test. Bruce protocol is widely used in treadmill exercise test and has been used in 66% of ordinary clinical tests (Evans & White, 2009; Schauer & Hanson, 1987). For treadmill exercise test, the participants were examined by a physician and pulse, blood pressure and ECG at the beginning and then repeatedly measured during the exercise test. Then the ECG electrodes were attached to the participants. The patients exercised on a treadmill. At any time the patient genuinely felt able to not exercise or if the patient has some symptoms such as chest pain, extreme shortness of breath, dizziness, severe headache, unusual changes in ECG or blood pressure, the exercise test was discontinued and maximal oxygen uptake was recorded.

### 2.2 Statistical Procedures

Data analysis was performed using SPSS 20. Independent samples t-test was run for both the control and intervention groups to compare the scores of risk perception, outcome expectancies, task self-efficiency, behavioral intention, action planning, coping planning, maintenance self-efficiency, recovery self-efficiency and family support, before and after intervention. To examine the impact of intervention on maintenance of physical activity and exercise capacity in CHD after discharge from rehabilitation, analysis of covariance was used. Past physical activity and base maximal oxygen uptake were used as covariate in the analysis. Preliminary examinations were done to ensure the assumptions of normality, linearity, homogeneity of variance, homogeneity of regression slope, and reliability of covariate.

## 3. Results

The mean age of participants was 57.28 in the range 41–76. They were mostly males (84.4% males and 15.6% females). The results of the Chi-square test for categorical variables and t-test for continuous variables (Table 1) showed that there was no significant difference between the two groups regarding demographic variables.

**Table 1:** Participants’ demographic characteristics, Chi-square test, and independent samples t-test for differences between the two groups

Variable	Control group number (%)	Intervention group number (%)	Total number (%)	χ^2^	P- value
Employment status				0.18	0.91
Housework	7(14.6)	8(16.7)	15(15.6)		
Employed	19(39.6)	20(41.7)	39(40.6)		
Retired	22(45.8)	20(41.7)	42(43.8)		
Education level				0.25	0.88
Preliminary	24(50.8)	22(45.8)	46(47.9)		
High school	11(22.9)	13(27.1)	24(25)		
University	13(27.1)	13(27.1)	26(27.1)		
Diagnosis				0.97	0.61
CABG	21(43.8)	22(45.8)	43(44.8)		
PTCA	15(31.2)	11(22.9)	26(27.1)		
MI	12(25.0)	15(31.2)	27(28.1)		
Sex				0.07	0.10
Male	41(85.4)	40(83.3)	81(84.4)		
Female	7(14.6)	8(16.7)	15(15.6)		

Age (year)	56.73(9.0)	57.83(8.71)		t=-0.61	0.54
Mean (SD)					

Results of independent samples t-test showed that there was no significant difference between the participants before intervention regarding risk perception, outcome expectancies, task self-efficiency, behavioral intention, action planning, coping planning, maintenance self-efficiency, recovery self-efficiency, family social support, past physical activity level and the amount of base maximal oxygen uptake. However, there was a significant difference after intervention. Table 2 shows a comparison of the mean and standard deviation of HAPA constructs, physical activity, and maximal oxygen uptake before and after intervention.

**Table 2:** Comparison of mean and standard deviation of HAPA constructs, physical activity, and maximal oxygen uptake before and after intervention

**Variable**	**Before intervention**			**After intervention**		

	Control group Mean (SD) (n=48)	Intervention group Mean (SD) (n=48)	P-value	Control group Mean (SD) (n=48)	Intervention group Mean (SD) (n=48)	P-value
Risk perception	16.94(1.56)	16.73(1.48)	0.51	16.15(1.68)	18.54(1.32)	<0.001
Outcome expectancies	33.48(1.91)	32.85(2.40)	0.16	31.69(1.80)	35.15(1.94)	<0.001
Task self-efficiency	9.88(1.33)	9.60(1.16)	0.29	9.00(0.96)	11.04(0.94)	<0.001
Behavioral intention	12.94(1.46)	12.81(1.56)	0.68	11.48(1.39)	14.42(1.44)	<0.001
Action planning	13.31(2.91)	12.69(3.02)	0.30	11.40(2.87)	15.02(2.45)	<0.001
Coping planning	12.48(2.77)	11.48(3.33)	0.11	10.42(2.63)	14.04(2.68)	<0.001
Maintenance self-efficiency	11.33(1.53)	10/77(1.72)	0.56	10.23(1.47)	12.48(1.45)	<0.001
Recovery self-efficiency	9.54(1.57)	9.17(1.46)	0.22	8.69(1.32)	10.69(1.09)	<0.001
Social support	14.54(3.89)	13.98(3.78)	0.47	13.17(2.39)	16.23(2.73)	<0.001
Physical activity	81.25(58.91)	70.10(62.09)	0.36	147.39(59.41)	182.86(110.21)	<0.001
**Maximal oxygen uptake**	**43.56(6.58)**	**43.30(6.69)**	**0.84**	**36.72(6.78)**	**42.54(6.44)**	**<0.001**

The analyses of covariance showed (Table 3) that after adjusting of pre-interventional scores, there was a significant difference between the two groups regarding their scores on the physical activity and maximal oxygen uptake after intervention.

**Table 3:** Adjusted mean and standard error of the participants’ physical activity and maximal oxygen uptake

Variable	Control group Mean (SE) (n=48)	Intervention group Mean (SE) (n=48)	F (1,94)	η^2^
Physical activity	144.72(12.16)	282.53(12.16)	66.68	0.41
Maximal oxygen uptake	36.62(0.60)	42.64(0.60)	49.11	0.34

## 4. Discussion

To the best of our knowledge, the present study is the first RCT, which has investigated the effect of an intervention based on HAPA together with family support in maintenance of physical activity and exercise capacity in CHD after rehabilitation. This study showed that HAPA-based intervention together with family support increases the scores of HAPA constructs and family support in the intervention group compared with the control group. In addition, the study showed the efficiency of HAPA-based intervention together with family support in the maintenance of physical activity and exercise capacity in CHD after rehabilitation. Consistent with the results of other studies on cardiac rehabilitation, the results of this study showed that intervention leads to initiation and maintenance of exercise in cardiac patients (Janssen, De Gucht, van Exel, & Maes, 2013; Meng et al., 2014). The results showed that intervention is effective in increasing both the physical activity and the maintenance of exercise capacity after rehabilitation. Four months after discharge from rehabilitation, 34% of the variance in maximal oxygen uptake and 41% of the variance in physical activity was explained by the intervention. These findings are comparable with the results of previous relevant studies. A study from Sniehotta, Scholz, & Schwarzer, (2006), that planning strategies were used to encourage patients to do physical activity 2 months after rehabilitation, demonstrated the participants in combined planning group (action planning plus coping planning) did significantly more physical activity compared with the other groups. The intervention was explained 9% of the variance in physical activity. In a comparable control group with the combined planning group, intervention was explained 12% of the variance in physical activity. Although in the present study physical activity was measured 4 months after rehabilitation a greater percentage of the variance was explained by the intervention due to the use of other approaches such as self-efficacy and family support may influence physical activity. As in another study conducted by Sniehotta et al. (2005), after cardiac rehabilitation and action control was added to planning intervention, they found long-term levels of exercise in the action control plus planning group had higher tendencies, although these differences were not significant. The study was conducted by Luszczynska (2006), in cardiac patients after myocardial infarction, demonstrated that the intervention led to greater use of planning strategies 8 months after myocardial infarction. Only patients who participated in an implementation intention intervention and used more planning strategies did physical activity 3 or more sessions per week, as recommended to them. The study from Lippke, Ziegelmann, and Schwarzer (2004), performed in orthopedic rehabilitation; recommended levels of action planning and exercise were higher in planning group than the control group. The results of these two studies are consistent with the results of the present study. In the present study, patients in the intervention group 4 months after the intervention, reported more planning and physical activity than the control group. In the present study, after interventions significant differences were observed between participants in the intervention and control groups in terms of HAPA constructs, family support, and physical activity level. Means for all variables significantly increased in the intervention group between pre-test and post-test. These findings were consistent with the study of Fleig, Pomp, Schwarzer, and Lippke (2013), which was performed in cardiac and orthopedic rehabilitation and showed the intervention led to an increase in planning, self-efficiency, and exercise after rehabilitation. Maximal oxygen uptake was reduced in both groups after intervention, the reason for this is that during rehabilitation three times a week, participants exercised regularly under the direct supervision of the rehabilitation team, but after discharge from rehabilitation exercise sessions per week was reduced. However, the rate of decline in maximal oxygen uptake was minimal in the intervention group and a significant difference in maximal oxygen uptake was observed in both groups after the intervention. Therefore, this study has been able to maintain the exercise capacity of participants in intervention groups 4 months after discharge from rehabilitation. These results are consistent with the results obtained from Giallauria et al. (2009), which was a multifaceted educational and behavioral intervention and lead to maintenance of maximal oxygen uptake in intervention group 2 years after rehabilitation in Myocardial infarction. Thus, these findings confirm the impact of HAPA-based intervention together with family support in maintenance of physical activity and exercise capacity in CHD after rehabilitation. The results of this study provide valuable information for health workers in secondary health settings and rehabilitation centers. Using the booklet designed based on HAPA together with family support is both cost-effective and useful.

## 5. Strengths and Limitations of the Study

Among the Strengths of this study, one may refer to simultaneous use of volitional and motivational factors in intervention and use of different self-regulation approaches and interpersonal factors. In addition, to measure the participants’ exercise capacity, an objective tool was used. Among the limitations of the study, the sample size was small and because all the participants simultaneously received intervention based on HAPA together with family support, efficiency of HAPA-based interventions with and without family support was not directly measured. Future studies should benefit from a design in which a group without family support is incorporated.
